# Crystal structure of E2814 bound to Tau

**DOI:** 10.1002/alz.094783

**Published:** 2025-01-09

**Authors:** Yu Chen, James Vance, Sara Jacob, Earl Albone, Shunsuke Ozaki, Corey Phillips, Mingde Shan, Tom Kazarian, Bona Poun, Hyeong‐wook Choi, Jared Spidel, James Staddon, Malcolm Roberts, John Wang

**Affiliations:** ^1^ Eisai, Cambridge, MA USA; ^2^ Eisai, Exton, PA USA; ^3^ Eisai, Hatfield United Kingdom

## Abstract

**Background:**

E2814 is a humanized monoclonal antibody that recognizes the microtubule‐binding region (MTBR) of tau, a region of the protein essential for filament formation and propagation in neurodegenerative diseases. Epitope mapping showed that E2814 binds to a specific sequence motif HVPGG in the MTBR. To elucidate the atomic interactions of E2814‐tau binding, we performed X‐ray crystallography studies with E2814 and various tau peptides containing the HVPGG motif.

**Method:**

The F_ab_ fragment of E2814 was incubated with 5 different tau peptides containing the 299‐HVPGG‐303 epitope at a 1:10 molar ratio prior to co‐crystallization screening. Co‐crystals of E2814/tau peptides were grown by sitting drop vapor diffusion, and the diffraction data were collected at synchrotron. Structures of E2814 bound to tau peptides were determined by molecular replacement.

**Result:**

Crystal structures of E2814 F_ab_ bound to five tau peptides containing 299‐HVPGG‐303 epitope were determined at 1.44 –1.64 Å resolution. The comparison of the five crystal structures revealed the sequence 299‐HVPG‐302 of tau presents the core region mediating recognition by E2814, which is also facilitated by I297 in the flanking region. The tau peptides are accommodated in the recognition groove formed by CDR regions from the VH and VL domains, and exhibited a U‐shaped conformation with close‐to quarter turns when bound to E2814. Both H299 and P301 at the turns mediate major interactions with E2814.

**Conclusion:**

Our results reveal the structural details of the key interactions between E2814 and the tau HVPGG motif, which adopts a U‐shaped conformation upon binding to E2814. These findings provide atomic insights into the mechanism of E2814‐mediated inhibition of tau aggregation and propagation.

